# Heterocellularity and Cellular Cross-Talk in the Cardiovascular System

**DOI:** 10.3389/fcvm.2018.00143

**Published:** 2018-11-01

**Authors:** Filippo Perbellini, Samuel A. Watson, Ifigeneia Bardi, Cesare M. Terracciano

**Affiliations:** Division of Cardiovascular Sciences, Myocardial Function, National Heart and Lung Institute, Imperial College London, London, United Kingdom

**Keywords:** cardiac fi broblast, endothelial cells (ECs), macrophages (M1/M2), multicellularity, myocytes, cardiac tissue, pericytes and vascular smooth muscle cells, inflammatory cell

## Abstract

Cellular specialization and interactions with other cell types are the essence of complex multicellular life. The orchestrated function of different cell populations in the heart, in combination with a complex network of intercellular circuits of communication, is essential to maintain a healthy heart and its disruption gives rise to pathological conditions. Over the past few years, the development of new biological research tools has facilitated more accurate identification of the cardiac cell populations and their specific roles. This review aims to provide an overview on the significance and contributions of the various cellular components: cardiomyocytes, fibroblasts, endothelial cells, vascular smooth muscle cells, pericytes, and inflammatory cells. It also aims to describe their role in cardiac development, physiology and pathology with a particular focus on the importance of heterocellularity and cellular interaction between these different cell types.

## Introduction

The development ofmulticellular organisms required millions of years of evolution, starting from simple prokaryotic cells, with no intracellular, or rudimentary organization, to eukaryotic cells, with more specialized, sophisticated cellular systems. Species evolved to include multiple specialized cells with distinct roles and functions. Populations of highly specialized cells form a variety of tissues, which allows the formation of organs capable of highly complex functions. Thus, multicellularity and the specialization of cells have driven evolution. The human body is one of the most studied multicellular systems and is comprised of more than 200 different cell types. Among these, the heart has been at the center of investigation not only because of its role in physiology but also because cardiac diseases are the number one cause of death in developed countries. The orchestrated function of different cell populations in the heart, in combination with a complex network of intercellular circuits of communication, is essential to maintain a healthy heart and its disruption gives rise to pathological conditions. Our knowledge of the precise factors involved in the orchestrated function and regulation of the heart is still incomplete. The different cellular components that form the heart, particularly the non-myocyte populations, have only recently been described in detail (1), making cardiac multicellularity a novel/topical target for cardiovascular research. Being at the center of the circulation, the heart is closely regulated by systemic and local signaling of chemical and mechanical nature, and this is also a very important area of investigation. Finally, the relentless electromechanical activity, which is unique to the heart, is also capable of regulating both cardiomyocyte and non-myocyte populations. This adds a crucial element of complexity that has limited our ability to investigate and understand cardiac behavior, particularly from the multicellular/heterocellular viewpoint. In this review, we will provide an overview of cardiac multicellularity and how both intercellular physical interactions and cell-cell signaling are fundamental in cardiac development and adult cardiac phenotype homeostasis.

### Cardiac multicellularity *in vivo*

**Figure 1:**
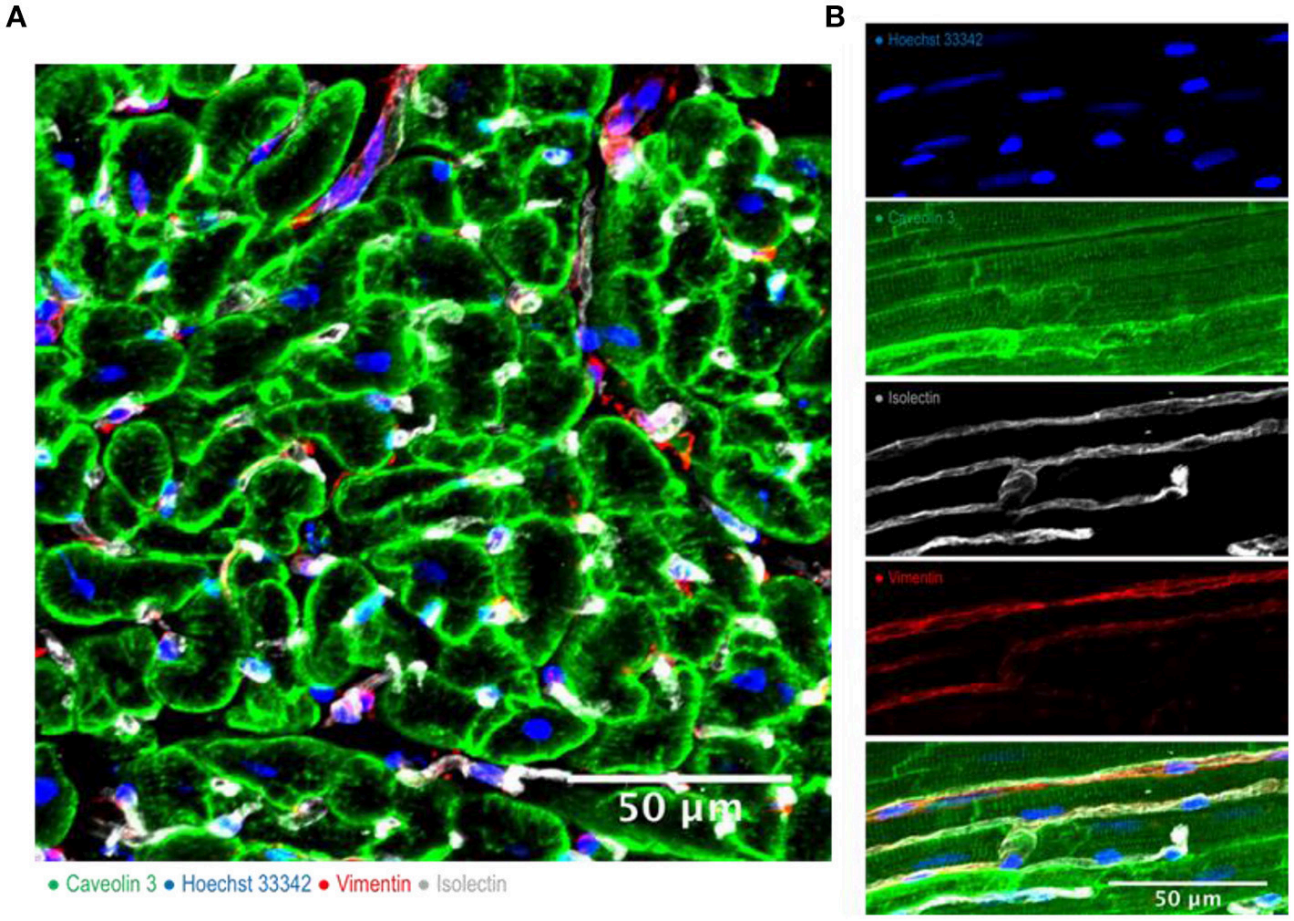
Cardiac multicellularity *in vitro*: Immunohistochemicalstaining and confocal microscopywere used to identify cardiac cells in a transverse section **(A)** and in a longitudinal section **(B)** of freshly prepared dog myocardial slices. Cardiomyocytes were labeled with caveolin 3, fibroblasts were labeled with vimentin and endothelial cells were labeled with isolectin. Nuclei were labeled with Hoechst 33342. Scale bar = 50 μm.

The heart is composed of several cell populations, each with specific functions and regulatory roles. Cardiomyocytes being very large cells make up most of cardiac tissue volume (2), but they only account for ≈25–35% of all the cells in the heart (3–5). Using genetic tools and cellular markers, it has recently been shown that endothelial cells make up >60% of the non-myocyte population, making them the most prevalent cell type in the adult heart (1) (Figure 1). A consensus is still lacking regarding the remaining stromal cell population composition. Previous studies (4, 6–9) have suggested that fibroblasts constitute the majority of non-myocytes, however, it is now known that they only account for <20% of the non-myocyte population (1, 5) (Table 1). Vascular Smooth Muscle Cells, pericytes, and hematopoietic-derived cells make up the rest of the non-myocyte population however a consensus on their respective percentage in cardiac tissue is still debated.

**Table 1:**
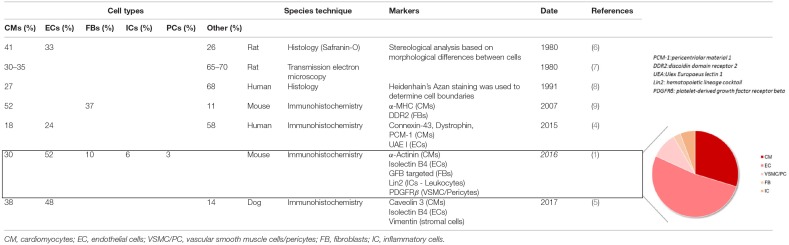
Cellular composition of the myocardium.

*CM, cardiomyocytes; EC, endothelial cells; VSMC/PC, vascular smooth muscle cells/pericytes; FB, fibroblasts; IC, inflammatory cells*.

## The role of cardiomyocytes

Cardiomyocytes are the muscle cells of cardiac tissue and their synchronous contraction is required to pump blood throughout the body. They are the most physically energetic cells in the body, repeating their relentless contraction cycle over 3 billion times in the average human lifespan (10). They are very large cells, typically 100–150 μm in length and 10–35 μm in width. Their cytoplasm is packed with sarcomeres, the contractile units of muscle cells, and mitochondria, which are needed to satisfy their high energy requirements and account for ~35% of cardiomyocyte volume (11). Cardiomyocytes are cylindrical in shape with end-to-end connections called intercalated disks. These highly specialized cell-to-cell connections ensure mechanical and electrochemical coupling (11). They help to stabilize the positions of the cells relative to each other and maintain the 3D structural integrity of the tissue (12). The intercalated disks are also the preferential method of cardiomyocyte cross talk. They contain intercellular channels called gap junctions, made of connexins. Ions, small molecules, and small peptides are capable of crossing these junctions. Disorganization of the intercalated discs can make gap junctions more susceptible to improper intercellular transfer of molecules and impulse propagation (12). The expression and distribution of junctional components are often altered in cardiovascular disease. It has been reported that mutations in the gene encoding connexin 40 GJA5 induce altered electric coupling and lead to increased arrhythmogenesis (13). Cardiac-specific loss of murine N-cadherin leads to a modest dilated cardiomyopathy with impaired cardiac function before sudden cardiac death (14). Cardiomyocyte regulation is also controlled by other cell types through paracrine signaling, however cardiomyocytes are also able to secrete soluble factors to interact and communicate with other cell types, particularly during inflammation or cardiac injury. A recent study by Roy et al. has shown that cardiomyocytes are also able to produce and secrete acetylcholine (ACh), a parasympathetic nervous system neurotransmitter. This non-neuronal source of ACh increases parasympathetic cholinergic signaling to counterbalance neural sympathetic activity regulating cardiac homeostasis and therefore plays a fundamental role in healthy heart activity (15). Inflammatory cytokines such as IL-6 are released by cardiomyocytes during hypoxic stress, suggesting an important role in the progression of myocardial dysfunction observed in cardiac ischemia-reperfusion injury (16). Although IL-6 has been reported to have cardioprotective effects (17), clinical studies suggest that prolonged and/or excessive synthesis of IL-6 is detrimental to the heart (18, 19). Cardiomyocytes have also been shown to produce and secrete TNF-α under certain conditions such as treatment with lipopolysaccharide (LPS). The presence of LPS contributes to the cardiovascular collapse and death observed in patients with sepsis. TNF-α stimulation on cardiomyocytes results in inotropic and pro-apoptotic effect which results in defective contractility and relaxation of the myocardium (20, 21). TNFα is another example of signaling molecule released by cardiomyocytes during myocardial infarction. TNFα release has been shown to be controlled by the hypoxia-inducible factor1α pathway and to be mediated by exosomes release by cardiomyocytes (22, 23).

## The role of endothelial cells

**Figure 2:**
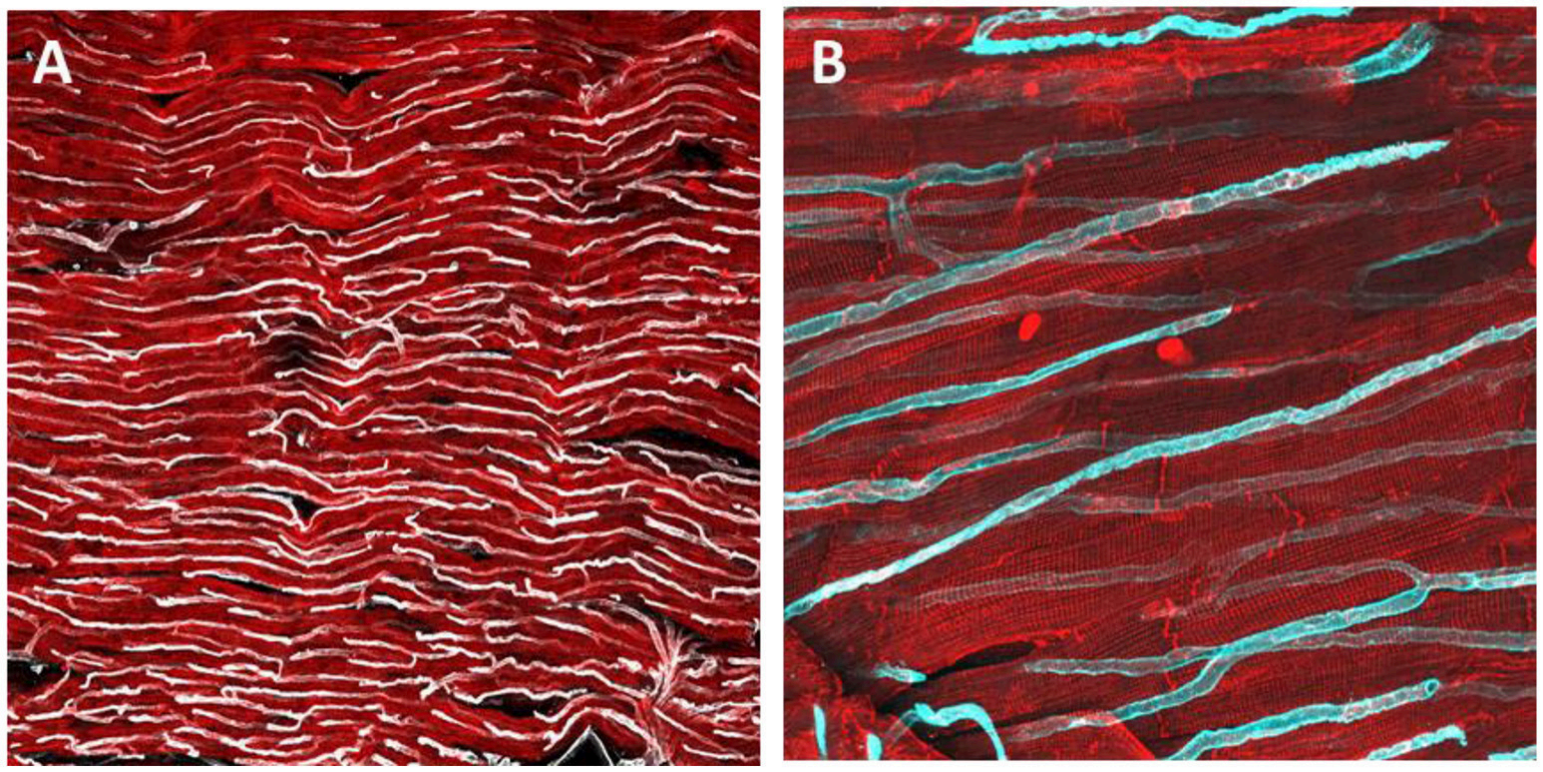
Immunohistochemical staining and confocal microscopy were used to identify endothelial cells distribution in a freshly prepared dog myocardial slice **(A)**. Higher magnification of capillaries and their location in proximity to cardiomyocytes **(B)**. Cardiomyocytes were labeled with caveolin 3 (red) and endothelial cells were labeled with isolectin (White and cyan).

In the healthy myocardium, a dense network of capillaries facilitates the distribution of oxygen and metabolic substrates to cardiomyocytes. Each cardiomyocyte is in contact with at least one capillary and endothelial cells outnumber cardiomyocytes by ≈3:1 (1, 24). This architectural arrangement also allows a mechanical and paracrine cross-talk between cardiomyocytes and endothelial cells to exist, which plays pivotal roles in cardiac development and the regulation of cardiomyocyte function (Figure 2). Several factors released by endothelial cells, including neuregulin, neurofibromatosis type 1 (NF1) and platelet-derived growth factor-B (PDGF-B), and by cardiomyocytes, including vascular endothelial growth factor-A (VEGF-A) and angiopoietin 1, have been implicated in these processes (25–27). Endothelial-cardiomyocyte interactions play fundamental roles in the regulation of cardiac function by both autocrine and paracrine mechanisms. Both endothelial cells and cardiomyocytes are able to synthesize Nitric Oxide (NO) with three different nitric oxide synthase isoenzymes (eNOS, iNOS, and NOS). eNOS expression in endothelial cells is four times greater than in cardiomyocytes (28). Nitric oxide affects blood vessels, its complex effect results in vasodilation due to relaxation of vascular smooth muscle, and reduced contractility in cardiomyocytes, leading to an attenuation of contraction (29). ET-1 is also released by endothelial cells, in addition to release by cardiac fibroblasts, and acts in both an autocrine and paracrine manner binding to cardiomyocytes via ET_A_ receptors and endothelial cells via ET_B_ receptors (24). ET-1 effects on cardiomyocytes include the induction of hypertrophy and remodeling. There is also evidence that endothelial cells promote cardiomyocyte survival, with neuregulin found to be a pro-survival factor (30) and less cardiomyocyte apoptosis observed when cardiomyocytes are cultured with endothelial cells *in vitro* (31).

The importance of endothelial factors during cardiac development has also been demonstrated by a number of cell-specific gene knockdown (KO) experiments. Mice lacking either neuregulin or its receptors, erbB2/4, die during mid-embryogenesis due to lack of cardiac trabeculae and cardiac cushion development (32). NF1 KO results in developmental defects in both the myocardial and endocardial cushions, resulting in myocardial thinning and ventricular septal defects. Defects do not occur in cardiomyocyte-specific KO models, indicating that signaling from endothelial cells is crucial for development (33). Endothelial-specific KO of PDGF-B results in cardiac abnormalities, including myocardial thinning, chamber dilation, hypertrabeculation, and septal defects, alongside vascular and glomerular abnormalities (34). Thus, molecular signals from endothelial cells are crucial for development but reciprocal cross-talk between cardiomyocytes and endothelial cells is also required. Mutations in both VEGF-A and its receptor, VEGF receptor-2, result in failure of both the endocardium and myocardium to develop. Cardiomyocyte-specific KO of VEGF-A results in defective angiogenesis and ventricular wall thinning (35). The angiopoietin-Tie-2 system is also fundamental to cardiac development and is primarily responsible for maturation and stabilization of the neovasculature (35). Mice with mutations in this pathway have an underdeveloped endocardium and myocardium, while cardiomyocyte-specific overexpression of angiopoietin-1 results in embryonic death (35). These findings demonstrate that sensitively controlled bilateral paracrine communication between endothelial cells and cardiomyocytes is fundamental to normal cardiac development.

## The role of cardiac fibroblasts

**Figure 3:**
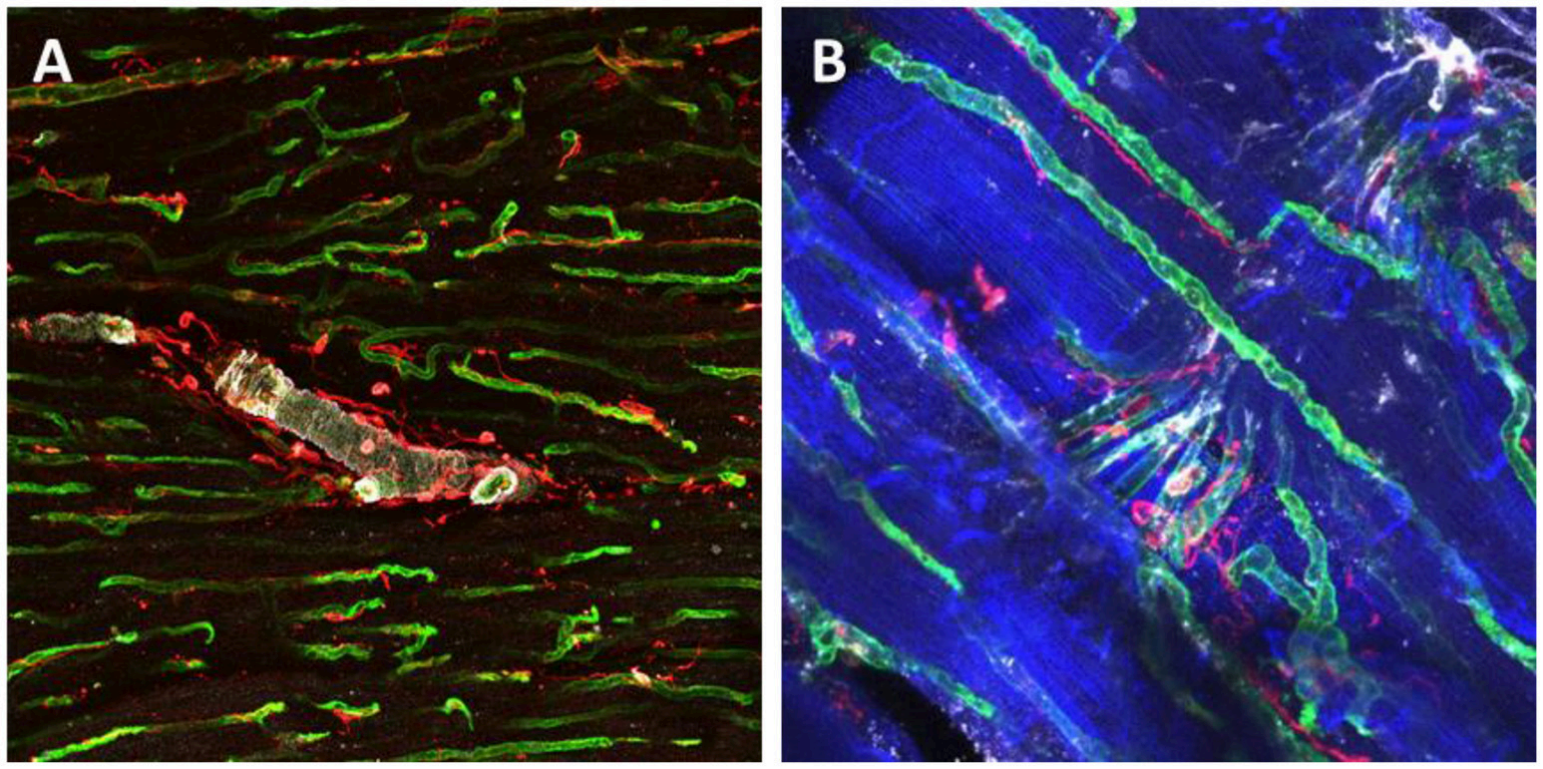
Immunohistochemical staining and confocal microscopy were used to identify different cardiac populations in a dog myocardial slice. Large vessels were identified for α-Smooth Muscle Actin expression (white), endothelial cells were labeled with isolectin (green) and fibroblasts were labeled with vimentin (red) **(A)**. Higher magnification of capillaries and their location in proximity to cardiomyocytes and fibroblasts **(B)**. Cardiomyocytes were labeled with caveolin 3 (blue), endothelial cells were double labeled with isolectin (green) and Von Willerbrand factor (white) and fibroblasts for Vimentin (red).

Cardiac fibroblasts are often considered the most abundant stromal cell type and they play a crucial role in extracellular matrix deposition, maintenance and remodeling. They are characterized by a secretory phenotype with an elongated, spindle-like morphology, a granular cytoplasm, and an extensive rough endoplasmic reticulum (36). In the heart they are diffusely distributed throughout the myocardium, localized in the interstitial space that separate cardiomyocytes and in close proximity to capillaries and larger vessels (Figure 3). To date, there is no agreement on appropriate markers to identify resident fibroblasts within the heart. The markers available (Vimentin, CD90, DDR2, FSP1, Sca1, Periostin, etc.) target different fibroblast-like cells suggesting that resting fibroblasts are a mixture of cell populations (5, 36). This hypothesis is further reinforced by the notion that cardiac fibroblasts come from two separate developmental origins. Two independent groups showed that fibroblasts residing in the interventricular septum and right ventricle do not form from the epicardium, but instead have an endothelial origin, constituting roughly 20% of the myocardial resident fibroblast (37, 38). Taken this into consideration, modern techniques of single cell analysis and genetic lineage tracing seem to show comparable gene expression profile of different cardiac fibroblast populations suggesting that they may not be as diverse as previously thought (37, 39).

The study of cardiac fibroblasts and their interactions with beating cardiomyocytes *in vivo* is problematic; fibroblast function is complex and multifaceted and there may be several direct and indirect mechanisms of cellular interactions. These include interaction via alteration of extracellular matrix (ECM) quantity and composition, vascular maintenance, paracrine signaling, conduction system insulation, and electrotonic coupling (36). The ECM is a complex, dynamic scaffold, composed of collagens, proteoglycans, and glycoproteins (10). Cardiac fibroblasts are involved in the synthesis and maintenance of the ECM and are responsible for the 5% turnover of the ECM each day (11). Cardiomyocytes are physically linked to the ECM via integrin molecules (12), allowing it to influence cardiomyocyte function through kinase signaling cascades and direct mechanical interaction with intracellular structures (13). As such, regulation of the ECM by fibroblasts indirectly influences cardiomyocytes. In addition to maintenance of the ECM, cardiac fibroblasts secrete a vast array of bioactive substances. These molecules are secreted into the interstitium, where they act in both an autocrine and paracrine fashion (40). The extensive array of soluble mediators released results in functional cross-talk between several cardiac cell populations, including cardiomyocytes. Of the numerous factors released, transforming growth factor beta (TGF-β), interleukin 6 (IL-6), and endothelin 1 (ET-1) have significant effects on cardiomyocytes. Secretion of TGF-β, typically induced by changes in mechanical loading (41), results in cardiomyocyte hypertrophy (42) and profound electrophysiological changes (43). At the whole heart level, these changes are initially protective, but ultimately result in maladaptive remodeling (44). IL-6 is also associated with cardiomyocyte hypertrophy, alongside diastolic dysfunction and reduced expression of SERCA2a (45). ET-1 induces a potent hypertrophy in cardiomyocytes and its expression directly correlates with ventricular remodeling (46, 47). Paracrine interactions can also be achieved via non-soluble mediators, such as extracellular vesicles and microRNAs (miRs). Fibroblast secretion of miR-21in exosomes has been shown to induce cardiomyocyte hypertrophy (48). The presence of all 3 isoforms of connexin (Cx40, Cx43, and Cx45) (49) and electronic coupling between cardiomyocytes and fibroblasts *in vivo* has been demonstrated using optogenetic techniques and tunneling nanotubes between the two cell types have also been observed (50). These findings have implications for cardiomyocyte electrophysiology and cardiac conductivity but the physiological importance of these interactions and their role in cardiac disease remains to be established.

A large variety of stimuli such as cytokines, cardiomyocyte death, or changes in mechanical load can activate cardiac fibroblasts into their pathological phenotype, known as myofibroblasts (40). Activated fibroblasts have a different morphology with increased cytoplasm, well-defined endoplasmic reticulum and Golgi complex and microfilament bundles, which are often identified with αSMA antibody. They also have altered functions which include decreased ECM degradation and excessive secretion of matrix proteins, including collagen Type 1, and pro-inflammatory cytokines which play a crucial role in scar formation and fibrosis (51). Previous studies have indicated that myofibroblasts can derive from pericytes, bone marrow progenitor cells, monocytes, and though endothelial to mesenchymal transition (51). The contribution of these various cell types is still debated, however, a recent and comprehensive study by Kanisicak et al. (39) using genetic lineage tracing, identified resident cardiac fibroblasts as the main source for activated myofibroblasts in the injured heart (39). Several studies have reported different roles of fibroblasts and myofibroblasts during physiology and disease in regulating myocardial function via soluble mediators (43). A study by Cartledge et al. have shown a smaller cardiomyocyte Ca^2+^ transient amplitude when cultured with myofibroblasts compared to fibroblasts suggesting an important bi-directional regulatory role of TGF- β (43). Similarly to fibroblasts, myofibroblasts are also capable of forming functional gap junctions in the diseased myocardium suggesting that myofibroblasts might contribute to arrhythmogenesis by direct electrotonic modulation of impulse propagation and increased mechanosensitive channel activation (41, 51–53).

## The role of vascular smooth muscle cells and pericytes

Vascular smooth muscle cells (VSMCs) are stromal cells and constitute the vascular wall of large and small vessels. By contraction and relaxation, these cells can alter the vessel luminal diameter and, as a consequence, they are responsible for the regulation of blood pressure and blood flow. VSMCs not only regulate vessel diameter for short periods but they can also be subjected to long-term stimulation which results in physiological (such as pregnancy or exercise) or pathological vascular remodeling (54). VSMCs are normally classified as contractile or synthetic. This simplification only represents the two ends of a spectrum which includes several intermediate phenotypes. These two cell types are different in terms of morphology, function, gene expression, marker profile, and gap junctional/adhesion molecules. Contractile VSMCs are elongated, spindle-shaped cells with contractile filaments and with a low proliferative and migratory capacity. Synthetic VSMCs on the other hand have a rhomboid or cobblestone morphology, high number of organelles and a high proliferative and migratory capacity (55, 56). Smooth-muscle myosin heavy chain (SM-MHC) and smoothelin are the two most common marker proteins used to identify contractile VSMCs, whereas syntethic VSMCs express Smooth-muscle-emb/non-muscle MHC and cellular retinol binding protein (CRBP-1) (54). Although VSMCs phenotype seems to be genetically programmed in relation to their developmental origin (57), as shown in different species such as rat (54), pig (58), and humans (59), local environmental stimuli can significantly modulate VSMCs characteristics and function. These includes physical as well as biochemical factors which act in combination with the extracellular matrix composition. Tensile stretch and shear stress, induced respectively by the blood pressure and blood flow, affecting VSMCs contractile state, can induce vessel wall remodeling (54). The cells of the endothelium are able to sense the shear stress and respond with nitric oxide release and with direct cell-cell interaction with the VSMCs (58, 60). Endothelial cell proliferation and dysfunction, associated with altered production of vasoactive mediators, such as nitric oxide, entothelin-1, serotonin and prostacyclin, are reported to alter VSMCs behavior and contribute to pulmonary arterial hypertension (61). PDGF molecules play a crucial role in cellular cross-talk, they are produced by endothelial cells, perivascular inflammatory cells and smooth muscle cells. During pulmonary arterial hypertension PDGF-A and PDGF-B are overexpressed, they induce fibroblasts activation and a synthetic phenotype in VSMCs with increased cellular proliferation and migration promoting pulmonary arterial remodeling (61, 62). TGF-β signaling is also involved in VSMCs regulation promoting a contractile phenotype on cultured adult smooth muscle cells (63). Recent studies revealed an important role of cellular cross-talk between macrophages and VSMCs and this phenomenon seems to plays an important role during atherosclerotic plaque formation. This communication, principally mediated by Toll-Like receptor pathways, can alter the ECM synthesis and deposition, increase the production of metalloproteinases and increase the production of angiogenic chemokines such as VEGF and IL-1 (57, 64, 65).

Pericytes are also an important contractile cell of the body. They are closely associated with the microvasculature, particularly with pre-capillary arterioles, capillaries, and post-capillary venules (65). Pericytes are normally embedded in the basal membrane in close contact with endothelial cells. In larger vessels of the myocardium, a sparse layer of pericytes separates the endothelium from the VSMCs and the elastic structures of the vessel (66). Morphologically they can be distinguished for their thin and elongated cytoplasm, numerous finger-like projections and the rounded nucleus (67, 68). A range of surface (PDGFRβ, CD146, CD13 and NG2) or cytoplasmic markers (αSMA, desmin, vimentin, and nestin) are commonly used to identify this specific cell population (68–70). The number of pericytes seems to be organ dependent and their number in cardiac tissue is still debated, with groups reporting pericytes to be the second most frequent myocardial cell with a ratio with endothelial cells of 2:1 or 3:1 (71). However, in light of more recent studies on cardiac cellular composition this numbers might be an overestimation (1, 5). If pericytes number is still uncertain, much more is known about their function. The cytoplasmic expression of contractile proteins, such as αSMA or vimentin, is a clear indication of their vasomotion regulatory role. Their main function is to regulate the homeostasis and permeability of the vasculature and to control the blood flow in the micro-circulation. They also play a role in the removal of cell debris and to monitor the maturation of endothelial cells (72, 73). Pericyte's cytoplasmic protrusions connect to cell membrane invaginations of endothelial cell though connexin43 mediated and N-Cadherin adherence junctions. These connections are used to sense mechanical forces, such as stretch and shear stress, and to exchange electrical (66) and biochemical signals (both ions and small molecules) (74, 75). The active cross-talk between pericytes and endothelial cells has been shown to be fundamental for the maintenance of the endothelial barrier, principally mediated by TGFβ and angiopoietin1 (76, 77) and the formation and deposition of collagen I, IV and fibronectin in the basal membrane (78). They also play an active role in the process of new vessel formation. They can induce quiescence and maturation in activated endothelial cells though Angiotensin I secretion (66) or bridge the temporary gaps formed between sprouting endothelial cells (66). In pathological conditions, particularly following ischemic damage, pericytes receive signals from resident cells and infiltrating inflammatory cells and play an active role in angiogenesis and collateralization, reparative fibrosis, tissue remodeling, and regeneration (66). It has been reported that macrophages secrete galectin-3 which stimulates pericyte's proliferation and secretion of protocollagen1 which eventually lead to collagen accumulation and cardiac fibrosis (66). Pericytes can also be activated followed injury via PDGF stimulation, which results in their migration to the interstitium, change into a myofibroblasts phenotype and increased release of ECM (66). During ischemia, cardiomyocytes release pro-Nerve Growth Factor which binds to the P75^NTR^ on pericytes inducing cytoskeletal changes, disrupting their interaction with endothelial cells and provoking vascular permability (66).

## The role of other cardiac cell populations

The immune cells form another important cardiac cell population. Of these, the role of macrophages has been most extensively explored over the last few years. Macrophages are an important component of the innate immune system and constitute a first line of defense against invading pathogens. They are large, round or spindle-like cells that contain a central round nucleus, have abundant clear, often vacuolated, cytoplasm with far-reaching protrusions, they are found in the interstitial space interspersed between cardiomyocytes, fibroblasts and endothelial cells (79). In the mouse heart it has been estimated that they can be up to 10% of non-cardiomyocytes cells and humans may have similar numbers (80). Following cardiac injury, an expansion of their population occurs through both local proliferation and monocyte recruitment, and is essential for myocardial repair (81). For several years macrophage heterogeneity was oversimplified into two main groups: M1 and M2. Macrophages that encourage inflammation are called M1, whereas those that decrease inflammation and encourage tissue repair are called M2 macrophages (35). Beyond having different functions, they also have distinct gene expression and surface markers profile (35). In the past few years it been shown that this classification does not adequately describe the spectrum of macrophage populations and several studies are now further investigating these differences (80, 82). The role of macrophages in the regulation of cardiomyocytes has been less well explored. A recent study by Hulsmans et al. has demonstrated that macrophages can form gap-junction with cardiomyocytes, via Cx43 expression, thus modulating their electrical activity (83). Furthermore, photo-stimulation of channelrhodopsin-2-expressing macrophages was able to improve atrioventricular conduction (83). Liu et al. (84) using a hypoxic mouse model and acyanotic vs. cyanotic patients, showed that postnatal hypoxia promoted cardiomyocyte proliferation and that cardiac resident macrophages may be involved in this process (35). Macrophages also communicate with other cell types in the myocardium, particularly with cardiac fibroblasts via the leucocyte surface antigen CD40 or the up-regulation of ICAM-1 and VCAM-1 (85, 86). Fibroblasts have the ability to participate in the maintenance of an inflammatory response via the expression of chemokines; on the other hand macrophages are the leading producers of TGF-β which is considered the most significant pro-fibrotic agent involved in the progression of chronic fibrotic diseases (87). It has long been recognized that macrophages can support angiogenesis, through both cell-to-cell contact with endothelial cells and the secretion of proangiogenic factors (88). Activated macrophages secrete a large variety of growth factors and inflammatory cytokines such as VEGF-A, VEGF-C, IL-1β, FGF2 etc., which induce endothelial activation, proliferation, spouting and survival (89, 90). Soluble proteases and matrix remodeling activity induced by macrophages also play a role in vessel sprouting and vascular growth (91, 92). Recent studies have shown that macrophages physically interact with sprouting endothelial cells to support and promote new vascular intersections in a process mediated by angiopoietin receptor, TIE2 and neuropilin-1 (88, 93). The interaction of macrophages and endothelial cells is bidirectional as endothelial cells can also promote the expansion and differentiation of proangiogenic macrophages. He et al. showed that endothelial cells can induce expansion and differentiation of hematopoietic progenitor cells toward an M2-macrophage phenotype (94). Gene marking studies, using Tie2-GFP reporter lentiviral vectors, frequently show clusters of immature Tie2-GFP+ cells monocytes in association with blood vessel sprouting (95, 96).

Although the interaction of macrophages with the other cardiac cell types has been quite extensively investigated, much less is known about the role of the other inflammatory cells (97, 98). Recent evidence suggests that T cells are also involved in the regulation of cardiac remodeling, particularly in the attenuation of hypertrophic response and cardiac dysfunction following myocardial infarction (99). TNFα overexpressing mice develop cardiomyopathy overtime, however the administration of anti-CD3 antibody to neutralize T cells reduced inflammatory cell recruitment and stopped hypertrophy (100). The depletion of T cells in Rag2 deficient mice, which develop pressure-overload induced hypertrophy, has also been shown to reduce macrophage infiltration and fibrosis together with attenuated cardiac dysfunction (101). Neutrophils are the most abundant leukocytes in humans and they also migrate to damaged areas following acute injury, such as myocardial infarction or ischemia. In literature very few studies can be found where their role has been investigated, particularly in hypertrophy and cardiac remodeling. A recent study from Wu et al. showed that the neutralization of S100A9, a molecule secreted by neutrophils, decreased angiotensin-II induced cardiac hypertrophy (102) suggesting a role of this cell type in cardiac remodeling. Several studies have also indicated an important role of T cells in the development of heart-specific autoimmune myocarditis (103–105). α-Myosin Heavy Chain specific (α-MyHC) CD4+ T cells have been found in mouse models of myocarditis as well as in human patients with inflammatory cardiomyopathy. Various CD4+ T cell subsets, particularly Th1 and Th17, have been shown to play an important role in the maintenance of tissue immune homeostasis and in modulating disease phenotypes (103–105). A better understanding of their role might therefore provide new approaches for the development of new therapeutic strategies. The number of mast cells in the heart also increases following cardiac injury. Mast cells have been implicated in maladaptive cardiac remodeling, but the mechanisms by which they contribute to this are yet to be fully elucidated (97). It has been shown that mast cells can release several bioactive molecules including growth factors, cytokines, histamine which affect other cell types (106–108). Changes in the concentrations of these factors can induce cardiomyocytes apoptosis as well as fibroblast proliferation and ECM deposition. Mast cell paracrine secretion of IL-4 has pro-fibrotic and immunomodulatory effects (106).

## Conclusions

Cellular specialization and interactions with other cell types are the essence of complex multicellular life. In recent years, the development of new research tools has enabled the identification of various cell populations within the myocardium. Interactions between different cell types in the heart have been identified as playing major roles in cardiac development and the maintenance of adult phenotype in both healthy physiology and pathological conditions. It is difficult to study cellular interaction *in vivo* and the data collected using current *in vitro* approaches are often oversimplified and do not recapitulate *in vivo* heterocellular complexity. Although progress is evident in the study of multicellularity and cellular interactions, key questions regarding multicellular interactions in an electromechanical stimulated environment remain to be answered. This review has summarized how chemical cross-talk can change cardiac cellular function. As the heart is subjected to electromechanical stimuli which affect cellular function, comprehensive studies and new models that incorporate mechanical, electrical as well as chemical signals need to be developed. New *in vitro* 3-dimensional heterocellular models that can recapitulate adult cardiac physiology are necessary in order to bridge the gap between existing *in vitro* and *in vivo* models. A more in-depth understanding of the role of cardiac microenvironment and heterocellular cross-talk is fundamental in the advancement of other research areas in cardiac biology, such as regenerative medicine and cardiac tissue engineering. The knowledge acquired will be fundamental to develop novel therapeutics with specific biological targets for treatments of patients with heart disease.

## Author contributions

FP wrote the first draft of the manuscript; SW and CT wrotesections of the manuscript. All authors contributed to manuscript revision, read and approved the submitted version.

### Conflict of interest statement

The authors declare that the research was conducted in the absence of any commercial or financial relationships that could be construed as a potential conflict of interest. The reviewer EA and handling editor declared their shared affiliation at the time of the review.
